# Subfailure Overstretch Induces Persistent Changes in the Passive Mechanical Response of Cerebral Arteries

**DOI:** 10.3389/fbioe.2015.00002

**Published:** 2015-01-28

**Authors:** E. David Bell, Jacob W. Sullivan, Kenneth L. Monson

**Affiliations:** ^1^Department of Bioengineering, University of Utah, Salt Lake City, UT, USA; ^2^Laboratory of Head Injury and Vessel Biomechanics, Department of Mechanical Engineering, University of Utah, Salt Lake City, UT, USA

**Keywords:** traumatic brain injury, stroke, sheep cerebral arteries, arterial softening, tissue damage

## Abstract

Cerebral blood vessels are critical in maintaining the health of the brain, but their function can be disrupted by traumatic brain injury (TBI). Even in cases without hemorrhage, vessels are deformed with the surrounding brain tissue. This subfailure deformation could result in altered mechanical behavior. This study investigates the effect of overstretch on the passive behavior of isolated middle cerebral arteries (MCAs), with the hypothesis that axial stretch beyond the *in vivo* length alters this response. Twenty nine MCA sections from 11 ewes were tested. Vessels were subjected to a baseline test consisting of an axial stretch from a buckled state to 1.05* *in vivo* stretch (λ_IV_) while pressurized at 13.3 kPa. Specimens were then subjected to a target level of axial overstretch between 1.05*λ_IV_ (λ_z_ = 1.15) and 1.52*λ_IV_ (λ_z_ = 1.63). Following overstretch, baseline tests were repeated immediately and then every 10 min, for 60 min, to investigate viscoelastic recovery. Injury was defined as an unrecoverable change in the passive mechanical response following overstretch. Finally, pressurized MCAs were pulled axially to failure. Post-overstretch response exhibited softening such that stress values at a given level of stretch were lower after injury. The observed softening also generally resulted in increased non-linearity of the stress-stretch curve, with toe region slope decreasing and large deformation slope increasing. There was no detectable change in reference configuration or failure values. As hypothesized, the magnitude of these alterations increased with overstretch severity, but only once overstretch exceeded 1.2*λ_IV_ (*p* < 0.001). These changes were persistent over 60 min. These changes may have significant implications in repeated TBI events and in increased susceptibility to stroke post-TBI.

## Introduction

In the United States, in 2009, there were an estimated 2.4 million cases of traumatic brain injury (TBI) (CDC, 2013). Annual estimates of death from TBI and stroke are 53,000 (Coronado et al., 2011) and 130,000 (Go et al., 2014), respectively, with corresponding treatment costs of 76.5 (CDC, 2013) and 36.5 (Go et al., 2014) billion dollars. Cerebral hemorrhage is a common outcome of TBI, but blood vessels that are not deformed enough to rupture and bleed may also be damaged. Such damage may progress to pathological conditions, including stroke (Chen et al., 2011; Hills et al., 2012; Burke et al., 2013) that significantly increase overall morbidity and mortality following TBI (CDC, 2013). A more complete understanding of vessel response to supra-physiological, subfailure deformations is needed to better define the progression of TBI.

The mechanical properties of cerebral vessels that experience large deformations may be altered. Blood vessels from other areas in the body have been shown to experience strain softening, a reduction in stress at a given level of strain, following overstretch in both the circumferential and axial directions (Holzapfel and Gasser, 2007; Alastrue et al., 2008; Horný et al., 2010; Peña et al., 2010; Maher et al., 2012a). The extent of softening following overstretch has been shown to be dependent on location within the arterial tree, possibly due to regional variations in the amounts of collagen and elastin (Maher et al., 2012b). Softening has also been observed in several other soft tissues, including ligament (Pollock et al., 2000; Quinn et al., 2007), tendon (Duenwald et al., 2009; Duenwald-Kuehl et al., 2012), and intestine (Gregersen et al., 1998). Several constitutive models have been proposed to predict softening behavior in blood vessels (Peña et al., 2010; Maher et al., 2012a; Weisbecker et al., 2012). Despite the potential value of these models, it has been noted that more experimental data are needed for these models to be useful for many regions of the vascular tree (Peña et al., 2010; Weisbecker et al., 2012).

Little has been done to explore softening in cerebral vessels. Accordingly, our objective was to characterize this phenomenon in cerebral arteries at various levels of overstretch relevant to TBI. Because these vessels commonly rupture and bleed with trauma, it is clear that they may experience deformations ranging from the physiological state to ultimate failure. While the detailed loading conditions they experience are yet undefined, the vessels are expected to align themselves with deformations imposed on the surrounding brain tissue. Therefore, alterations of cerebral artery mechanical properties resulting from a large range of axial overstretch values were explored. We hypothesized that even small levels of overstretch would alter vessel response but that the magnitude of the imposed changes would increase with extent of overstretch. Changes in properties were also evaluated over time following overstretch to differentiate persistent changes from temporary viscoelastic alterations.

## Materials and Methods

### Sample acquisition and preparation

A total of 29 middle cerebral artery (MCA) sections were dissected from 11 adult ewes. All procedures met requirements established by the Institutional Animal Care and Use Committee at the University of Utah. Eight of the animals were pregnant Columbia-Rambouillet ewes, and the remaining three (Columbia ewes) were obtained from a local slaughterhouse. Pregnant ewes were euthanized via an overdose of Beuthanasia (MWI Veterinary Supply, Boise, ID, USA), and the brain was immediately removed from the skull and placed in 5°C Hanks buffered saline solution (HBSS; KCl 5.37, KH_2_PO_4_ 0.44, NaCl 136.9, Na_2_HPO_4_ 0.34, d-Glucose 5.55, NaHCO_3_ 4.17; concentrations in mM) for transport back to the lab. Slaughterhouse ewes were euthanized via a humane stunner applied to the occipital lobe (not in the vicinity of the MCA tested), and the head was placed on ice for transport back to the lab where the brain was immediately removed and placed in 5°C HBSS until testing. Brains from slaughterhouse animals were removed from the skull within 2 h of death. As in previous studies (Bell et al., 2013), branches were ligated, vessels were secured to stainless steel needles attached to the testing apparatus, and black glass beads were spread over the adventitia to be used as fiducial markers (GL-0760, size 25–53 μm, MO-SCI Specialty Products, Rolla, MO, USA). Lack of calcium in HBSS ensured a passive response. All samples were tested within 48 h of death (Humphrey, 1995; Stemper et al., 2007; Amin et al., 2011; Weisbecker et al., 2013).

### Experimental apparatus and methodology

The mechanical testing apparatus was similar to that described previously (Bell et al., 2013). Briefly, the displacement of the needles on which the MCAs were mounted was controlled using a vertical custom linear stage (Parker Automation, Cleveland, OH, USA). Vessels were perfused with HBSS, and luminal pressure was controlled through a syringe attached to a computer-controlled linear actuator (D-A0.25-AB-HT17075-4-P, Ultra Motion, Cutchongue, NY, USA). Axial load and pressure were measured with an inline 250 g capacity load cell (Model 31 Low, Honeywell, Golden Valley, MN, USA) and pressure transducers (26PCDFM67G, Honeywell, Golden Valley, MN, USA) corresponding to the vessel inlet and outlet, respectively. Actuator positions were given by digital encoders (resolution 1.0 μm). The specimen was bathed in HBSS during testing, and its deformations were monitored with a digital video camera (PL-A641, Pixelink, Ottawa, ON, Canada) attached to a zoom lens (VZM 450i, Edmund Optics, Barrington, NJ, USA). Test control and data acquisition were performed with a custom LabVIEW program (National Instruments, Austin, TX, USA).

Following mounting of the MCA, it was preconditioned by oscillating the luminal pressure (6.7–20 kPa; 50–150 mmHg) for five cycles while length was held constant. Preconditioning tests were repeated at gradually increasing lengths until the *in vivo* length was identified (Van Loon et al., 1977). A final preconditioning test was conducted at a stretch level of 1.05 times the *in vivo* length. Each MCA was then subjected to an unpressurized axial stretch test to determine its unloaded length.

Following preconditioning, a five step protocol was conducted. *Step 1:* undamaged, baseline response was defined through a quasi-static, axial stretch test to ~1.05*λ_IV_. *Step 2:* vessels were subjected to a single quasi-static, axial overstretch to one of six target levels ranging from 1.05*λ_IV_ (control vessels) to 1.5*λ_IV_. Overstretch values were chosen to explore the full range of vessel axial stretch possible during TBI. Each overstretch group included 4–6 specimens. *Step 3:* to investigate any viscoelastic recovery in the sample after overstretch, each vessel was subjected to a baseline response test (Step 1) immediately following overstretch and then every 10 min for 60 min. *Step 4:* following the final repeated baseline test, an unpressurized axial stretch test to the previous maximum overstretch level was conducted to determine any changes in the unpressurized stress-stretch response. *Step 5:* finally, the vessel was axially stretched to failure. Luminal pressure was maintained at 13.3 kPa throughout the procedure, except during Step 4 as described.

### Data analysis

Data collected during experiments were processed as previously described (Bell et al., 2013). Interpolation was used to produce one-to-one correspondence between current diameter (*d_e_*) measurements obtained from video at 3 Hz and other data collected at 100 Hz. Reference diameter (*D_e_*) and cross sectional area (*A*) were measured from unloaded vessel cross-sections cut from the end of each sample. Reference inner diameter (*D_i_*) was computed from these values. Current inner diameter (*d_i_*) during tests was calculated by assuming incompressibility (Eq. 1).

(1) $$d_{i}=\sqrt{d_{e}^{2}-4A∕\left(\pi \lambda_{z}\right)}$$

Axial stretch (λ_z_) was determined separately using actuator displacements and adventitial marker displacements, with reference length *L* taken at the unloaded configuration in both cases. Subsequent analysis showed that axial stretch values obtained from marker positions were highly variable, particularly during tests with high overstretch levels (see Discussion). As a result, reported axial stretch values are based on actuator position only.

Vessels were assumed to be homogeneous circular cylinders, with mid-wall stretch defined by Eqs 2 and 3

(2) $$\begin{array}{l} \lambda_{\theta }=\left(\frac{d_{i}+d_{e}}{D_{i}+D_{e}}\right) \end{array}$$

(3) $$\begin{array}{l} \lambda_{z}=\left(\frac{l}{L}\right) \end{array}$$

where subscripts θ and *z* refer to the local cylindrical coordinates in the circumferential and axial directions, respectively. Enforcing equilibrium in the two directions results in the mean Cauchy stresses defined in Eqs 4 and 5

(4) $$\begin{array}{l} T_{\theta }=p_{i}\left(\frac{d_{i}}{d_{e}-d_{i}}\right) \end{array}$$

(5) $$\begin{array}{l} T_{z}=\frac{\lambda_{z}}{A}\left(F_{z}+\frac{\pi }{4}p_{i}d_{i}^{2}\right) \end{array}$$

where *F_z_* represents the experimental axial force, and *p_i_* is the luminal pressure. Residual stresses were not considered.

**Figure 1:**
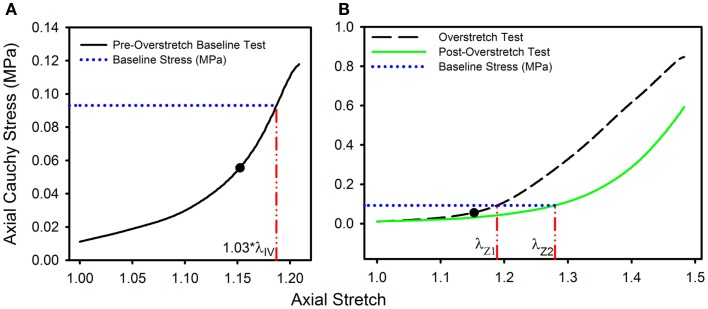
Data from a representative sample showing the definitions of (A) the baseline stress from the initial pre-overstretch baseline test (at 1.03*λ_IV_) and (B) the baseline stretch levels λ_Z1_ (from the overstretch test; λ_Z_ max = 1.3*λ_IV_) and λ_Z2_ (from the post-overstretch failure test; cropped data shown) corresponding to the baseline stress. (●) indicates the undamaged *in vivo* stress-stretch state.

To quantify the effect of overstretch, five parameters derived from the pre- and post-damage stress-stretch curves were compared: *in vivo* stiffness, tare load stretch, baseline stretch, strain energy, and failure values. *In vivo* stiffness was calculated as the slope of the curve at *in vivo* stretch in pressurized axial stretch tests (Monson et al., 2008; Bell et al., 2013). Tare load stretch was defined as the stretch value associated with an axial load of 0.0005 N, a force value consistently outside the noise range of the load cell, intended to help identify possible changes in reference length. Additionally, baseline stress was defined as the axial stress level corresponding to 1.03*λ_IV_ in the pre-overstretch baseline test (Figure 1A). The corresponding stretch level was defined as the baseline stretch and was quantified in both the overstretch test (λ_Z1_) and the post-overstretch failure test (λ_Z2_) since both always included the baseline stress level (Figure 1B). The initial measurement of baseline stress was taken at 1.03*λ_IV_ rather than the maximum applied stretch (1.05*λ_IV_) in order to avoid any artifacts in the data caused by deceleration of the actuator near the peak stretch level. The percent change in axial stretch for both the tare load stretch and the baseline stretch was calculated using (Eq. 6).

(6) $$\%\Delta \lambda_{z}=100*\frac{\lambda_{2}-\lambda_{1}}{\lambda_{1}}$$

where λ_1_ and λ_2_ are the relevant pre- and post-damage stretch values. Similar to work by others (Maher et al., 2012b), softening was also quantified using percent change in strain energy (%ΔU) (Eq. 7), where *A_1_* and *A_2_* are the areas under the curve to the overstretch level before and after damage, respectively, except in the case of repeated baseline tests where the maximum stretch was 1.05*λ_IV_.

(7) $$\%\Delta U=100*\frac{A_{1}-A_{2}}{A_{1}}$$

One way ANOVA, followed by a two-tailed *t*-test with a Bonferroni correction, was used to determine the significance of differences between groups, with *p* < 0.05 indicating statistical significance for ANOVA. *Post hoc* tests between groups required a *p*-value below 0.00625 for significance. Additionally, data acquired from the pregnant and slaughter house ewes were compared using analysis of covariance (ANCOVA) to determine if there was any statistical difference between the groups.

## Results

**Figure 2:**
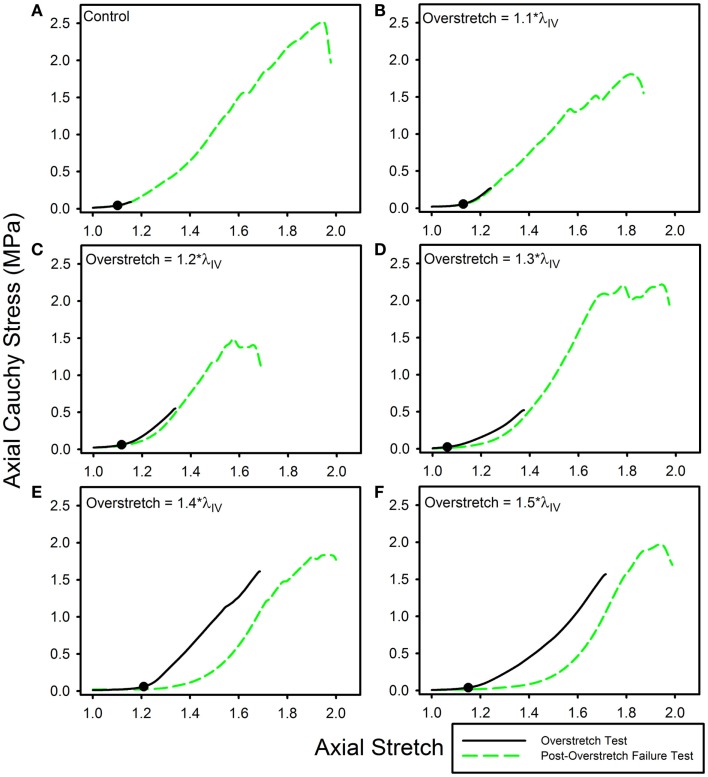
Data from representative samples showing axial stress-stretch responses for overstretch tests and post- overstretch failure tests for the (A) control vessels, (B) 1.1*λ_IV_ overstretch group, (C) 1.2*λ_IV_ overstretch group, (D) 1.3*λ_IV_ overstretch group, (E) 1.4*λ_IV_ overstretch group, and (F) 1.5*λ_IV_ overstretch group. Note: there is increased softening as the overstretch applied increases. (●) indicates the undamaged *in vivo* stress-stretch state.

Twenty nine arteries were successfully tested. Mean (±SD) unloaded length and outer diameter of these specimens were 3.63 (±0.58) and 0.98 (±0.09) mm, respectively. Mean axial *in vivo* stretch was 1.12 (±0.04). The undamaged axial response of the arteries was qualitatively similar to what we have previously reported for other cerebral arteries (Figure 1A) (Monson et al., 2008; Bell et al., 2013). Post-overstretch response exhibited softening such that stress values at a given level of stretch were lower after injury (Figure 2). The observed softening also generally resulted in increased non-linearity of the stress-stretch curve, with toe region slope decreasing and large deformation slope increasing. There was no detectable change in reference configuration or failure values. As hypothesized, the magnitude of change increased with overstretch severity.

**Figure 3:**
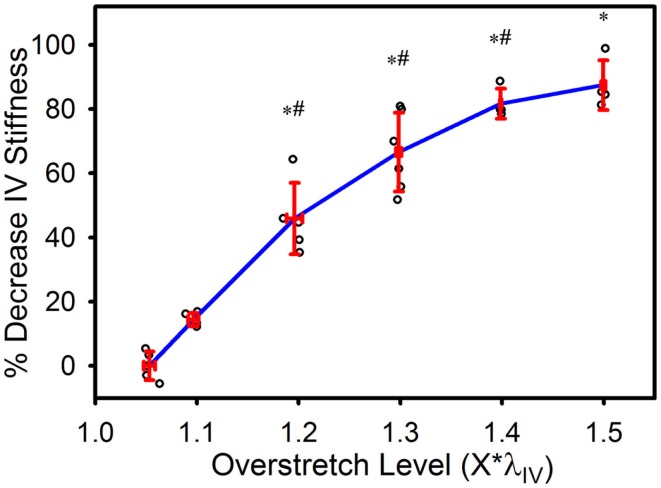
**Percent decrease in axial *in vivo* stiffness following overstretch**. Red error bars indicate SD for each group. Blue line connects group means to clarify trends. (○) indicates individual data points. (*) indicates statistical difference from pre-overstretch values. (**#**) indicates statistical difference from the group subjected to an overstretch two levels lower.

*In vivo* axial stiffness was shown to decrease with overstretch (Figure 3). However, this change in stiffness was non-linear, with less difference between adjacent groups with higher overstretch. Mean pre-overstretch *in vivo* stiffness was 0.65 (±0.13) MPa. *In vivo* stiffnesses from the control (non-overstretched) and 1.1*λ_IV_ overstretch groups were not significantly different from the pre-damage value (Table 1). However, the measure was significantly reduced following each of the higher overstretch levels, with reductions of 40 and 80% following overstretches of 1.2 and 1.5, respectively. None of the large overstretch groups was found to be different from the adjacent group at a lower stretch level. However, the 1.3*λ_IV_ (*p* < 0.001) and 1.4*λ_IV_ (*p* = 0.0058) groups were statistically different from the overstretch group two levels lower. The 1.5*λ_IV_ group was not significantly different from the 1.3*λ_IV_ group (*p* = 0.16).

**Table 1:** **Statistical summary for *in vivo* stiffness (IV stiffness), tare load stretch, baseline stretch, and percent change in strain energy (%ΔU) following various levels of overstretch, including mean ± SD, number of samples, and *p*-value for comparison between overstretched and undamaged groups**.

	Undamaged	Overstretch level					
		Control	1.1	1.2	1.3	1.4	1.5
IV stiffness (MPa)	0.65 ± 0.13 (*n* = 29)	0.70 ± 0.17 (*n* = 5, *p* = 0.42)	0.57 ± 0.12 (*n* = 5, *p* = 0.09)	0.36 ± 0.07 (*n* = 5, ***p****** < 0.001***)	0.18 ± 0.06 (*n* = 6, ***p****** < 0.001***)	0.13 ± 0.03 (*n* = 4, ***p****** < 0.001***)	0.07 ± 0.05 (*n* = 4, ***p****** < 0.001***)
Tare load stretch	1.01 ± 0.03 (*n* = 23)	No data	1.03 ± 0.02 (*n* = 5, *p* = 0.17)	1.01 ± 0.02 (*n* = 4, *p* = 0.90)	1.05 ± 0.04 (*n* = 6, *p* = 0.02)	1.11 ± 0.04 (*n* = 4, ***p****** < 0.001***)	1.12 ± 0.02 (*n* = 4, ***p****** < 0.001***)
Baseline stretch (λ_*z*_)	1.03 ± 0.001 (*n* = 28)	1.03 ± 0.005 (*n* = 5, *p* = 0.86)	1.04 ± 0.001 (*n* = 5, *p* = 0.88)	1.06 ± 0.006 (*n* = 4, ***p****** < 0.001***)	1.11 ± 0.02 (*n* = 6, ***p****** < 0.001***)	1.15 ± 0.007 (*n* = 4, ***p****** < 0.001***)	1.18 ± 0.03 (*n* = 4, ***p****** < 0.001***)
%ΔU	N/A	−1.19 ± 10.5 (*n* = 5)	11.12 ± 1.84 (*n* = 5, *p* = 0.01)	32.88 ± 6.03 (*n* = 4, ***p****** < 0.001***)	49.26 ± 8.63 (*n* = 6, ***p****** < 0.001***)	56.55 ± 3.14 (*n* = 4, ***p****** < 0.001***)	59.72 ± 6.71 (*n* = 4, ***p****** < 0.001***)

*Significant *p*-values are bolded*.

**Figure 4:**
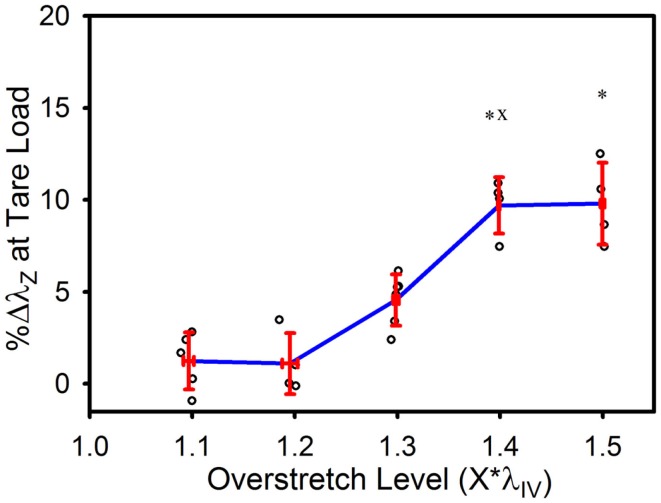
**Percent increase in tare load stretch following overstretch**. Red error bars indicate SD for each group. Blue line connects group means to clarify trends. (○) indicates individual data points. (*) indicates statistical difference from pre-overstretch values. (x) indicates statistical difference from the adjacent group subjected to a lower overstretch level.

The tare load stretch was also affected by overstretch, but only once a threshold was surpassed, as shown in Figure 4. Mean tare load stretch in undamaged vessels was λ_*z*_ = 1.01 (± 0.03) (Table 1). Stretch values were not statistically different from this value in overstretch groups below 1.3*λ_IV_ (note that this measure was not explored in controls other than undamaged specimens). Tare load stretch in the 1.4*λ_IV_ and 1.5*λ_IV_ groups was significantly increased relative to the pre-overstretch mean, but the two groups were not statistically different from each other (*p* = 0.59). Also, the 1.4*λ_IV_ (*p* = 0.003) group was the only group statistically different from the adjacent group subjected to a lower overstretch.

**Figure 5:**
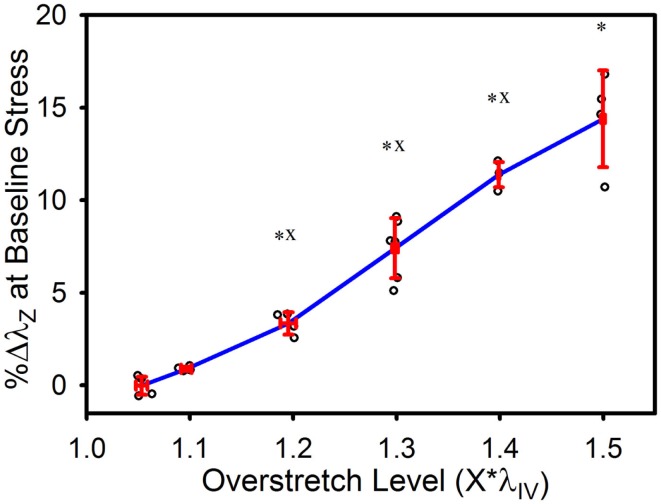
**Percent increase in axial stretch, as measured at the baseline stress level, following overstretch**. Red error bars indicate SD for each group. Blue line connects group means to clarify trends. (○) indicates individual data points. (*) indicates statistical difference from the pre-overstretch mean baseline stretch. (x) indicates statistical difference from the adjacent group subjected to a lower overstretch level.

As illustrated in Figure 5, the baseline stretch increased with overstretch. Measurements from the 1.1*λ_IV_ group were not significantly different from controls, but each of the higher overstretch groups demonstrated significance (Table 1). The 1.2*λ_IV_, 1.3*λ_IV_, and 1.4*λ_IV_ groups were all significantly different from their adjacent lower group (*p* < 0.001). Statistical analysis also showed a difference between the 1.4*λ_IV_ and 1.5*λ_IV_ groups (*p* < 0.0001), but there was concern about the higher variance in the 1.5*λ_IV_ group, despite the baseline stretch data, as a whole, passing the Levene’s test for the equality of variances for the groups (*p* = 0.09 > 0.05). Subsequently, a basic two-tailed *t*-test was conducted solely between the 1.4*λ_IV_ and 1.5*λ_IV_ groups resulting in *p* = 0.11, suggesting that the groups were not statistically different. Accordingly, the more conservative of these two statistical results was used in this case, leading to the conclusion that this particular difference was not significant. Finally, the baseline stretch measured from the overstretch tests (prior to reaching peak overstretch) was not statistically different from the initial baseline measurement (*p* = 0.95, *n* = 28).

**Figure 6:**
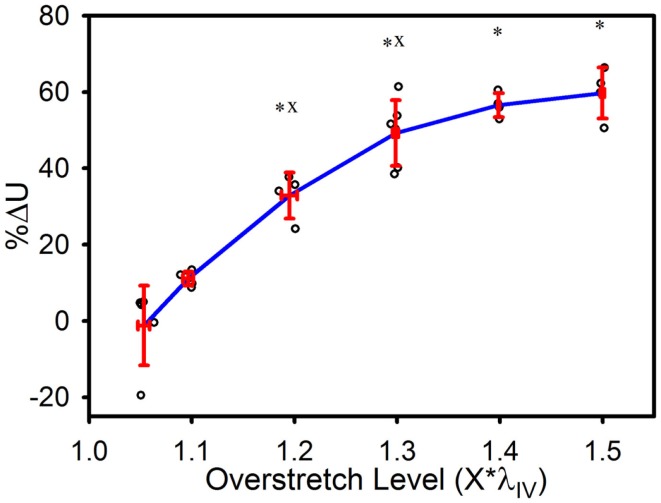
**Percent decrease in strain energy under the axial stress-stretch curves, as calculated from initial overstretch test data, and post-overstretch failure test data (data truncated to stop at the previous overstretch level)**. Red error bars indicate SD for each group. Blue line connects group means to clarify trends. (○) indicates individual data points. (*) indicates statistical difference from the control (non-overstretched) group. (x) indicates statistical difference from the adjacent group subjected to a lower overstretch level.

The strain energy was also affected by overstretch, with increasing levels of overstretch leading to larger reductions in strain energy, similar to the pattern of change observed with *in vivo* stiffness. As shown in Figure 6, the percent decrease, relative to the control group, was not significantly different in the 1.1*λ_IV_ group but was significant for all higher overstretch levels (Table 1). Further, the overstretch groups 1.2*λ_IV_ (*p* = 0.001) and 1.3*λ_IV_ (*p* = 0.012) were different from the adjacent lower overstretch group. However, once overstretch exceeded 1.3*λ_IV_, differences between adjacent groups were no longer significant, similar to the pattern observed with *in vivo* stiffness. It should be noted (Figure 6) that there is a particular data point in the control group that is far lower than all the others, indicating a physically unreasonable increase in strain energy in the post-injury (sham) axial stretch test. Due to small axial forces at low stretch levels, the luminal pressure has a relatively large influence on the total axial stress (Eq. 5). Thus, it is likely that this unexpected data point is due to a slight variation in luminal pressure between the first and second stretch tests used to calculate %ΔU. This effect from pressure, and subsequent larger deviation in the control data, could also be the reason for the lack of significance in the differences between the control and 1.1*λ_IV_ overstretch groups.

**Figure 7:**
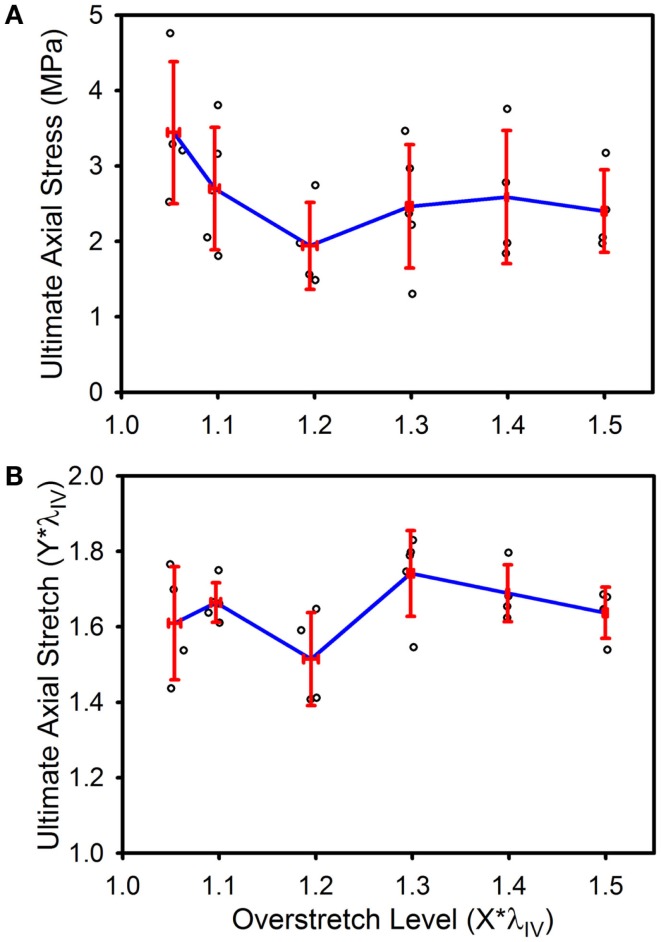
**Ultimate stress (A) and stretch (B) for the various overstretch groups, as measured from the final pressurized axial stretch test (pulled to failure)**. Red error bars indicate SD for each group. Blue line connects group means to clarify trends. (○) indicates individual data points.

Ultimate stress and stretch values were not significantly affected by the level of imposed overstretch (Figure 7). Only those samples which failed within the midsection were used to quantify the failure properties. Control (*n* = 4) ultimate failure stress and stretch were 3.44 (±0.94) MPa and 1.61*λ_IV_ (±0.15*λ_IV_) (or λ_*z*_ = 1.73 ± 0.21), respectively. Ultimate stress showed a tendency to decrease with overstretch, but the trend was not statistically significant.

**Figure 8:**
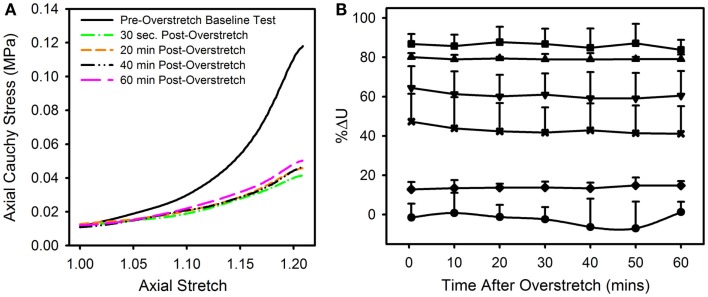
**(A)** Data from a representative sample showing the axial stress-stretch curve for the pre-overstretch baseline test, as well as four of the seven post-overstretch baseline tests which were repeated every 10 min for 60 min after overstretch. **(B)** Means and SD for the various test groups and how these values evolved over time. Note, there was no significant recovery of strain energy following overstretch at any of the tested overstretch levels. [Symbols: (● Control Group, *n* = 5), (♦ 1.1*λ_IV_ Group, *n* = 5) (**x** 1.2*λ_IV_ Group, *n* = 5), (▼1.3*λ_IV_ Group, *n* = 6), (▲ 1.4*λ_IV_ Group, *n* = 4), (■ 1.5*λ_IV_ Group, *n* = 4)].

The observed changes appear to be enduring, rather than passively recoverable due to viscoelasticity (Figure 8A). In order to measure any time dependence in the observed changes, the strain energy from the repeated baseline tests was calculated and compared to that of the pre-overstretch baseline test (Figure 8B). While the magnitude of the %ΔU following overstretch increased as the imposed overstretch increased, it did not change significantly over the 60 min it was measured. Preliminary tests extended this time frame up to 6 h without a change in the result.

In order to determine if there was any effect in the data imposed by either the source of the tissue or the time from death, ANCOVA tests were conducted on the change of baseline stretch following overstretch, as this was the most sensitive metric taken. Accordingly, it was determined that the data acquired from the two tissue sources (pregnant ewes vs. slaughter house ewes) were not significantly different (*p* = 0.498). Also, data sets were divided into bins defined by how long after death the vessels were tested (<8, 8–22, 25–44 h after death). These bins also roughly coincide with differing amounts of time the samples were refrigerated. This comparison also showed no significant effect in the data (*p* = 0.313).

## Discussion

The present study aimed to characterize stretch-induced softening in cerebral arteries. Results show that axial overstretches increased the amount of stretch required to obtain a target level of stress. Overstretch also reduced strain energy and *in vivo* stiffness. These effects were only significant above a certain threshold of overstretch. There was no recovery of properties over a 60-min period. These changes may have important implications in repeated TBI events and in increased susceptibility to stroke post-TBI (Chen et al., 2011; Hills et al., 2012; Burke et al., 2013).

In order to see how overstretch would alter the mechanical behavior of cerebral arteries at physiological levels, both *in vivo* stiffness and baseline stretch were determined. The *in vivo* stiffness quantifies how the physiological stiffness of the vessel would change following overstretch, assuming the surrounding brain tissue returned to a similar configuration after injury. This stiffness value was significantly affected by overstretches above 1.2*λ_IV_, with a decrease in stiffness of over 40% at 1.2*λ_IV_. The baseline stretch allows estimation of vessel length under similar loading after an injury. Given the observed decrease in stiffness, it is not surprising that baseline stretch was also significantly affected by overstretch, starting at the same threshold (1.2*λ_IV_). These two metrics together show that the mechanical behavior of the lower end of the mechanical response is dramatically altered by even mild levels of overstretch. Since it has been shown that a higher stiffness in arteries is associated with improved stability (Cyron et al., 2014), it is reasonable to theorize that the inverse would also be true. Thus, these observed low end mechanical changes could cause the cerebral vessels to be more susceptible to subsequent pathologies, including stroke and aneurysm.

The mean tare load stretch prior to injury coincided with ~1% axial strain, or λ_*z*_ = 1.01 (±0.03). As a result, the percent change in tare load stretch used here is comparable to metrics referred to in previous studies as either permanent set (Peña et al., 2010; Peña, 2011) or residual inelastic strain (Alastrue et al., 2008; Maher et al., 2012a,b; Weisbecker et al., 2012). In the current study, increased levels of overstretch resulted in an increase in the tare load stretch, similar to a previous arterial softening study by Maher et al. (2012b). However, as the goal of Maher et al. was to quantify differences between different arteries, they did not report statistical differences between loading levels applied to the same artery type. In the present study, the change in tare load stretch did not become significant until overstretch exceeded ~1.3*λ_IV_ to 1.4*λ_IV_. The change in tare load stretch then ceased to significantly change beyond the 1.4*λ_IV_ overstretch level. In contrast, Maher et al. (2012b) observed a linear increase in residual inelastic strain up to the highest tested overstretch, corresponding to λ_*z*_ = 1.6 (or ≈1.45*λ_IV_) in this study. While this level of overstretch was lower than the highest level investigated here, the magnitude of change in tare load stretch at 1.4*λ_IV_ for the cerebral arteries falls between the magnitudes of inelastic residual strain measured in carotid and femoral arteries reported by Maher et al. (2012b). This is consistent with their conclusions that this effect would be increased in more muscular arteries. This change in tare load stretch may indicate a change in the unloaded reference configuration or may just be a result of a reduction of slope in the stress-stretch curve between the reference and tare load configurations.

Strain energy was significantly affected by overstretch. The metric %ΔU quantifies the progression of damage averaged over the entire overstretch range. Interestingly, the magnitude of %ΔU ceases to significantly change once the overstretch level exceeds 1.3*λ_IV_. Thus, it is possible that some aspect of microstructural damage is mostly complete by this overstretch level. A previous study of arterial softening investigated a similar parameter, referred to as “% stress softening” (Maher et al., 2012b), where the upper limit of the area quantified was a common axial force rather than a common level of stretch. This difference likely describes why Maher et al. (2012b) did not observe any significant change in strain energy with overstretch, while we did. We limited the analysis of strain energy to the region bounded by the previously applied overstretch level since subsequent loading clearly showed additional damage prior to reaching the previous peak load. This damage behavior was observed in the form of temporary drops in axial stress as the stretch continued to increase toward ultimate failure, as seen in the failure curve of (Figure 2E).

It is interesting to note that significant changes were seen in the *in vivo* stiffness, baseline stretch, and strain energy as low as the 1.2*λ_IV_ level. The range at which these various measurements of damage are significantly altered coincides well with a previous study of microstructural damage in rabbit aorta, which showed that repeated strains corresponding to 1.3 times *in situ* length resulted in a significant increase in microstructural damage relative to their control group (Austin et al., 2010).

The observed decreasing level of change in the various metrics at higher overstretch levels has not been previously reported in experiments, and the cause for it is not entirely clear. However, it is interesting to note that this phenomenon appears to be predicted by a constitutive damage model that includes both continuous and discontinuous softening (Peña et al., 2009). In this model, the inclusion of continuous damage resulted in an overall “damage” parameter having a decreased change at higher levels of stretch. The parameter did not have such a decreasing change when only considering discontinuous damage. This is due to the fact that the contribution from continuous damage in their model could reach a saturation point associated with a certain stretch level, where any additional damage at higher stretches would have increasing levels of discontinuous damage but a constant contribution from continuous damage. As the data in the current study does not show roll-off in the measured changes until overstretch levels approach failure, the application of such a damage model to these data would require a continuous damage saturation parameter that would ensure this contribution would level off near, yet prior to, ultimate failure. Comparison to models like this could lend insight into microstructural changes responsible for the softening phenomena observed here.

Interestingly, ultimate failure values were not significantly affected by overstretch. While the literature is lacking in comparable data for arteries following overstretch, these results are similar to what has been observed in ligaments (Panjabi et al., 1996). Panjabi et al. investigated failure values in undamaged and overstretched samples. They showed that the axial force and displacement, as well as the “energy to failure,” were unchanged after an overstretch to 80% of failure (approximately corresponding to the 1.3*λ_IV_ stretch level in this study). While the current study did not measure strain energy to failure, it is suspected, based on the softening observed at the lower end of the mechanical response that strain energy to failure would be reduced following overstretch in the ewe MCAs. However, it is perhaps less surprising that the failure properties would be unchanged provided there was no gross damage applied to the sample beforehand. The ultimate stress and stretch in a fibrous biological tissue would be associated with the highest values that can be sustained when all the fibers are maximally aligned in the direction of stretch. Any stretch beyond the point where fibers actually begin to fail on a large scale would naturally support less stress, thus occurring after the point where the ultimate stress was measured. Accordingly, it is logical to suspect that the overstretch-induced changes in this study are primarily due to rearrangement of the microstructural components rather than large-scale ruptures in the fibrous components, but additional analysis is needed to relate damage and microstructural change.

We did not observe any significant recovery of mechanical properties over the hour between overstretch and failure tests. Previous studies of viscoelastic recovery in tendons and ligaments show significant recovery in as little as 100 s (Duenwald et al., 2009, 2010). Maher et al. (2012a) discussed preliminary data investigating viscoelastic recovery in porcine arteries over a period of 1–2 h, without observing significant recovery. They suggested that a much longer period of time could be needed for recovery. While this is certainly possible, our preliminary studies showed no quantifiable recovery for 6 h post-overstretch.

Arterial softening has been shown to be dependent on location in the vascular tree (Maher et al., 2012b), but this is the first study known to quantify this effect in cerebral arteries. These location-specific differences are theorized to be due to variations in the proportions and arrangement of collagen and elastin fibers making up the arterial microstructure (Peña et al., 2010; Maher et al., 2012b; Weisbecker et al., 2013). The structure of cerebral arteries is different from other muscular arteries, as is evidenced by the lack of an external elastic lamina (Lee, 1995). Previously, Maher et al. (2012b) tested several non-cerebral arteries with varying proportions of collagen and elastin in the vessel wall. As mentioned briefly above, they subsequently concluded that overstretch of more muscular arteries results in greater inelastic strains. Their results agree well with the measurement of tare load axial stretch in this study.

Given the significance of location in the vascular tree, it should be noted that the present study was conducted on sheep MCA, though TBI-induced deformations are likely more relevant in smaller vessels. Previous work from our lab has shown that the microstructure and mechanical properties of human MCA and pial arteries are very similar (Monson et al., 2005). As a consequence, it seems reasonable to expect similar softening patterns for the two vessels. Use of the MCA here is also helpful for comparison with different animal models of both TBI and stroke where the MCA characterization is common (Högestätt et al., 1983; Coulson et al., 2002, 2004; González et al., 2005; Bell et al., 2013). Trauma-induced softening is likely also important in smaller arteries and arterioles, as well as in veins, venules, and perhaps even capillaries, but findings from this study are not expected to translate directly to these various vessel types due to significant microstructural differences.

As noted in the Section “Materials and Methods,” black glass beads were placed on the adventitial surface in order to track local strain. However, the stretch values measured from markers were often inconsistent with observed overall vessel motion. For example, when samples were overstretched to relatively high overstretch levels, it was common to see a decrease in marker-derived axial stretch well before the overall overstretch was concluded. Based on this observation, it is likely that local sites of damage did not correspond with marker placement in a way that allowed reliable measurement of strains corresponding to that damage. During testing at high overstretch values, local sites of damage were observed to occur unpredictably at various locations throughout the specimen. Measurements from markers could not then be expected to yield consistent measurements of deformation. As a result, average stretch values were calculated from actuator displacement in order to provide a more consistent basis for analysis between specimens.

There are some potential limitations to the current study. First, while the human system is of primary interest to our group, these tests were conducted in sheep cerebral vessels. However, overstretch-induced softening behavior has been observed in human systemic arteries (Weisbecker et al., 2012, 2013). Also, preliminary testing in human cerebral arteries has confirmed the presence of softening behavior in these vessels, though a detailed characterization has not been performed (Monson, 2001). So while the data in the current study are not directly translatable to human tissue, it does provide a greater understanding of the progression of softening in cerebral vessels associated with overstretch.

Second, while the majority of the sheep MCAs were obtained from pregnant ewes, some were acquired from non-pregnant ewes through a local slaughterhouse. It has been shown (Griendling et al., 1985) that arteries close to the fetus during pregnancy can have altered mechanical properties. However, this same study showed that arteries further away, such as the carotid arteries, do not have any pregnancy-induced changes in the mechanical properties. It was reasoned for this study that since the MCA is further along the arterial tree than the carotid arteries, it would be similarly unaffected by pregnancy. As reported, statistical testing showed no difference between the two groups.

The third limitation has to do with the quasi-static rate used in the present study. Deformations associated with TBI take place at a high rate. This quasi-static loading rate was chosen in order to enable comparison to pre-damage mechanical behavior (Monson et al., 2008; Bell et al., 2013). Further, the quasi-static loading rate allowed for more precise control of the levels of overstretch applied to samples in this study. Mechanical properties of cerebral vessels have been shown to be relatively insensitive to loading rates ranging from 0.001 to 50 s^−1^ (Chalupnik, 1971) and 0.01 to 524 s^−1^ (Monson et al., 2003). However, the possibility exists that the progression of softening may be altered at higher loading rates. This should be explored further.

The final limitation is related to the fact that specimens were stored at 5°C prior to testing. The duration of this refrigerated storage ranged from a few hours to as much as 40 h. It has been observed that refrigeration of tissue prior to testing can potentially alter material properties (Stemper et al., 2007; Chow and Zhang, 2011). However, refrigeration of arterial tissue samples prior to testing is a common practice (Humphrey, 1995; Amin et al., 2011; Maher et al., 2012b) and has been determined by other studies to have little effect on both the active and passive properties of arteries, with up to 48 h of storage at 5°C (Herlihy and Murphy, 1973; Cox, 1978; Dobrin, 1984). While Stemper et al. (2007) showed that subfailure stresses and the ultimate failure stress can be lower following refrigeration than in fresh tissue samples, subfailure strains, and ultimate failure strains were not significantly different between fresh and refrigerated tissues in their study. As all of our samples were similarly refrigerated, if there was any refrigeration-induced effect on the failure properties, it would be expected to have been similar for all samples, thus, not changing the relationship of the failure properties between overstretch groups. Finally, as mentioned above, no statistical difference was found for refrigeration time.

Overall, results demonstrate that sheep cerebral arteries experience a persistent change in properties as a result of isolated overstretch. It is currently unknown whether similar changes occur *in vivo*, but it is known that the brain and its supporting vasculature are deformed during trauma. While TBI is complex, including a number of factors that could promote blood vessel dysfunction, changes in vessel mechanical properties would likely play an important role in any acute, and potentially chronic, vessel impairment. Further work is needed to more fully characterize changes induced by overstretch. In particular, this study focused on changes in axial behavior, but alterations to circumferential response are surely also important physiologically and should be explored. However, even without direct study of circumferential changes, multiaxial considerations suggest that the observed increase in axial laxity will lead to a less stiff response circumferentially. At least in an isolated vessel, these changes could lead to disturbances in blood pressure and flow due to both buckling and circumferential expansion. It is not yet clear how forces from surrounding tissue may influence this, especially in an injured brain, but changes to vessel properties are likely an important consideration in the progression of TBI.

Because vessels likely do not experience axial stretch alone during head trauma, additional work is also needed to define the influence of circumferential overstretch on properties in both directions. While this information is expected to lead to a better understanding of TBI, it may be even more useful in improving surgical interventions. Finally, the vasculature is not passive, with both active regulation of its circumference (through smooth muscle cell contraction) and remodeling of the wall to adapt to both physiological and mechanical changes. Future investigation of how these active processes are affected by, or how they impose an effect upon, softening would also be beneficial.

## Conflict of Interest Statement

The authors declare that the research was conducted in the absence of any commercial or financial relationships that could be construed as a potential conflict of interest.
